# A new experiment on the use of images to answer web survey questions

**DOI:** 10.1111/rssa.12856

**Published:** 2022-05-20

**Authors:** Oriol J. Bosch, Melanie Revilla, Danish Daniel Qureshi, Jan Karem Höhne

**Affiliations:** ^1^ London School of Economics and Political Science London UK; ^2^ Research and Expertise Centre for Survey Methodology (RECSM) Universitat Pompeu Fabra Barcelona Spain; ^3^ Cheil Germany Frankfurt Germany; ^4^ Collaborative Research Center SFB 884 “Political Economy of Reforms” University of Mannheim Mannheim Germany

**Keywords:** breakoff, images, motivational messages, noncompliance, smartphone, web survey

## Abstract

Images might provide richer and more objective information than text answers to open‐ended survey questions. Little is known, nonetheless, about the consequences for data quality of asking participants to answer open‐ended questions with images. Therefore, this paper addresses three research questions: (1) What is the effect of answering web survey questions with images instead of text on breakoff, noncompliance with the task, completion time and question evaluation? (2) What is the effect of including a motivational message on these four aspects? (3) Does the impact of asking to answer with images instead of text vary across device types? To answer these questions, we implemented a 2 × 3 between‐subject web survey experiment (*N* = 3043) in Germany. Half of the sample was required to answer using PCs and the other half with smartphones. Within each device group, respondents were randomly assigned to (1) a control group answering open‐ended questions with text; (2) a treatment group answering open‐ended questions with images; and (3) another treatment group answering open‐ended questions with images but prompted with a motivational message. Results show that asking participants to answer with images significantly increases participants' likelihood of noncompliance as well as their completion times, while worsening their overall survey experience. Including motivational messages, moreover, moderately reduces the likelihood of noncompliance. Finally, the likelihood of noncompliance is similar across devices.

## 1 INTRODUCTION

During the last few years, web surveys have been increasingly answered with smartphones (Bosch et al., 2019b; Peterson, 2012; Revilla et al., 2016). The enhanced capabilities of smartphones provide new methodological opportunities. In particular, smartphone sensors and apps allow researchers to collect new types of data, which can improve and expand survey measurement (Link et al., 2014), and offer the potential to reduce measurement errors, respondent burden and data collection costs (Jäckle et al., 2018). For example, GPS (McCool et al., 2021), accelerometers (Höhne & Schlosser, 2019; Höhne, Revilla, et al., 2020), web tracking applications and plug‐ins (Bosch & Revilla, 2021b, 2022; Revilla et al., 2017) and microphones (Gavras & Höhne, 2022; Revilla & Couper, 2021; Revilla et al., 2020), have already been used in (mobile) web survey research.

Images, in particular, still represent a mostly unexplored opportunity for survey research. Within the survey context, images can come from (1) a photo taken with the camera from the participants' devices during the survey, (2) an image already stored by the participants and shared with the researchers during the survey or (3) even a screenshot of some information displayed on the screen of the device (see Iglesias & Revilla, 2021). Although an exhaustive list of conditions of when images can be useful for survey research has yet to be developed, there are several contexts in which asking for images has already been tested as an alternative that could improve data quality. First, images have been used to collect objective data that could be affected by high measurement errors when measured with survey questions. For instance, Ohme et al. (2020) measured smartphone usage by asking participants to take and upload a screenshot of their iOS Screen Time app. Second, images have also been proposed as a substitution for complex survey tasks (e.g. survey batteries), potentially reducing participants' burden. For example, Jäckle et al. (2019) asked participants to scan receipts to collect expenditure data, which had the potential of substituting multiple and lengthy questions about the products bought and their price tags. Finally, images have been used to gather information that participants might not be aware of and, hence, could lead to inaccurate reports or ‘don’t know' answers. For instance, Ilic et al. (2020) asked participants to upload a photo of their heating system, to gather information about the type of systems in use, which most participants might be unaware of. Beyond questions of data quality, answering with images could also help to make the survey experience feel more natural, interesting and enjoyable for participants (van Heerden et al., 2014) since sharing images has become one of the most popular online activities (Madden et al., 2013). Finally, computer vision algorithms, which can help researchers automatically extract information from images (e.g. labels, text recognition), are evolving rapidly (Mulfari et al., 2016). This can potentially make processing visual data easier and more affordable (Bosch et al., 2019a).

Despite high hopes on the potential opportunities to collect richer data with images (Bosch et al., 2019a), concerns have been raised about the potential negative effects of these innovative data collection approaches on a variety of other aspects, such as breakoff and compliance rates, completion times and the overall survey experience (e.g. Bosch & Revilla, 2021a; Revilla et al., 2020). Little is known, however, about the impact of asking participants to answer survey questions with images on these aspects. Therefore, in this study, we address the following research questions (RQ):

**RQ1.** What is the effect of answering open‐ended survey questions with images instead of typing in text on the following four aspects: breakoff, compliance with the task, completion time and how participants evaluate the questions (i.e. the extent to which participants like answering the questions and find them easy)?

Even if the causal link between data quality indicators and answering with images has not been determined yet, past research shows that both the stated willingness to share images in the frame of web surveys (12.4%–18.7% depending on the type of images in Struminskaya, Lugtig, et al., 2021; 18%–38% depending on the type of images in Struminskaya, Toepoel, et al., 2021; 50% in Revilla et al., 2019; 65% in Wenz et al., 2019) and the actual compliance (29%–43% depending on the type of images in Ilic et al., 2020; 59% in Bosch et al., 2019a, 2019b) are rather low. There is the need, therefore, to develop strategies to increase compliance rates. Although recent research has already tested some strategies with limited success (e.g. assuring confidentiality or saying that images would reduce the number of questions; Struminskaya, Lugtig, et al., 2021), the inclusion of motivational messages that remind participants that their answers are important and valuable has yet to be investigated. Hence, our second research question is:

**RQ2.** What is the effect of including a motivational message (compared to not including one) on the same four aspects (breakoff, compliance with the task, completion time and how participants evaluate the questions)?

Finally, since PCs and laptops are frequently equipped with cameras and the required software for accessing and sharing stored images, asking for images in the context of surveys should not be limited to web surveys conducted on mobile devices. Nonetheless, the little evidence available about the feasibility of doing so is exclusively focused on mobile devices. Considering that PCs and mobile devices differ on several aspects, such as screen size, keyboard type or the context in which surveys are answered, the impact of asking for images on breakoff, noncompliance, completion time or how participants evaluate the questions might differ between PCs and smartphones. If this is the case, the available evidence might not necessarily apply to web surveys conducted on PCs. Hence, we explore a third research question:

**RQ3.** Does the impact of asking participants to answer with images instead of text on the same four aspects vary between PC and smartphone respondents?

## 2 BACKGROUND

### 2.1 Impact of answering survey questions with images instead of typing text

Previous research provides some information about the impact that answering survey questions with images might have on breakoff and noncompliance rates. On the one hand, using information from a non‐probability web survey of Millennials, Bosch et al. (2019a) found that 53% of participants uploaded a photo taken in the moment and 59% uploaded an already saved image when asked to do so. These results are similar to the 56% rate of compliance that Ribeiro and Kuegler (2014) found for LightSpeed panellists. On the other hand, in the context of the Dutch general population probability‐based panel Longitudinal Internet studies for the Social Sciences (LISS), Ilic et al. (2020) show that asking participants to answer with images instead of text leads to a reduction of compliance rates of between 31 and 70 percentage points, depending on the task. Considering this, we present the following hypotheses:

Asking participants to take a photo or upload an image compared to asking them to type an answer increases the participants' likelihood of breakoff (*H1a*) and noncompliance (*H1b*).

Regarding the impact of answering with images on completion time and how participants evaluate the questions, there is no specific evidence yet available. However, some research has already studied how completion time and survey evaluation are affected when asking respondents to answer with new types of data compared to typing answers. For instance, Bosch and Revilla (2021a) found that asking Millennials to answer with emojis increased completion times (in Mexico and Spain) and the proportion of participants who liked answering the experimental questions (in Spain). Conversely, Revilla et al. (2020), exploring the impact of asking participants to answer with voice tools (e.g. recording), found a significantly lower proportion of participants liking the survey or finding it easy to answer, as well as a significantly lower completion time, compared to those who were asked to type their answers. Considering that taking a photo or finding an image and uploading it is more demanding than both answering with emojis and using voice tools, we propose the following hypotheses:

Asking participants to take a photo or upload an image compared to asking them to type in an answer increases completion times (*H1c*) and reduces the likelihood of participants liking the questions and finding them easy (*H1d*).

### 2.2 Impact of motivational messages

Previous research has studied the possibility of motivating respondents to increase their participation rates and decrease breakoff and item nonresponse rates by providing specific messages in the introduction (Revilla, 2016) or within the course of the survey (Sakshaug & Crawford, 2010). While some have found null effects when showing generic or personalised motivational messages before or within a survey question with regard to breakoff or item nonresponse (Kapelner & Chandler, 2010; Sakshaug & Crawford, 2010), others have found that these messages sometimes work. For example, Al Baghal and Lynn (2015), using data from the Innovation Panel (UK), found that prompting a respondent with a motivational message after a missed question decreased item nonresponse. Moreover, Bosch et al. (2018), analysing survey experiments showing several motivational messages through the surveys in waves 2, 4 and 6 of the probability‐based CROss‐National Online Survey (CRONOS), consistently found a small reduction of item nonresponse in the UK, but no effect in Estonia and Slovenia. Taking these findings into account, we propose the following hypotheses:

Adding a motivational message when asking to answer with an image reduces the participants' likelihood of breakoff (*H2a*) and noncompliance (*H2b*).

Including motivational messages can also have an impact on other indicators, such as completion times or survey evaluation. On the one hand, motivational messages included in the CRONOS panel increased completion times for participants in Estonia (Wave 6 only) but reduced them for participants in Slovenia and the UK (Bosch et al., 2018). On the other hand, motivational messages included in CRONOS were found to have non‐significant small effects on the reported survey enjoyment and difficulty. Hence, we present the following hypotheses:

Adding a motivational message when asking participants to answer with an image has no significant effect on completion times (*H2c*) and the likelihood of participants liking the questions and finding them easy (*H2d*).

### 2.3 The interaction with device

Ample research has explored whether PC and smartphone respondents differ in terms of our four aspects of interest. Overall, evidence shows that smartphone participants present higher breakoff and item nonresponse rates than PC respondents (e.g. the meta‐analysis by Couper et al., 2017; Mavletova & Couper, 2015) and completion times (Couper et al., 2017, p. 360), but similar survey evaluations (Revilla, 2017). In the context of asking for images, nonetheless, other factors can also play a role. Individuals, for instance, are less familiar with the procedure of uploading images from their PCs than from their smartphones (Mendelson & Papacharissi, 2010). Carrington (2020) reported that, by 2020, 91% of all pictures taken worldwide would come from a smartphone camera. This implies that (a) individuals are more used to taking pictures with their smartphones and (b) most pictures are saved on smartphones. Hence, when asked to answer with images, participants using smartphones might find it easier to perform the task of taking a photo and/or finding a suitable image. Considering this, we present the following hypotheses:

The negative impact of asking participants to answer with images on breakoff (*H3a*), noncompliance (*H3b*), completion times (*H3c*) and the extent to which participants like the questions and find them easy (*H3d*) is lower for smartphone than for PC participants.

## 3 METHOD AND DATA

### 3.1 The experiment

To test our hypotheses, we designed a 2 × 3 between‐subject web survey experiment. First, we randomly invited panellists to participate in the survey either through PCs (*PC*) or smartphones (*S*). At the start of the survey, participants who did not comply with the device requirement were not allowed to continue participating and were asked to use the correct device. Those who used the correct device were allowed to continue, while those not complying were not. Since the size of each device subsamples was fixed and quotas were set at the general sample level and not at the device subsample level, this caused a self‐selection of individuals, which lead the sample composition to differ significantly across the PC and smartphone subsamples in terms of the main demographic variables. Appendix A contains information on the sample composition of the different experimental groups. For those participants who used the correct device, in the second step and within device groups, we randomly assigned them to one of the following three groups: (1) a control group answering open‐ended questions using a traditional text entry format (*Text*), (2) a treatment group answering the questions with images (*Image*) and (3) another treatment group answering the questions with images but including the following motivational message after the question (*ImagePush*): ‘Your collaboration is very important for our research and will help us to improve your experience with surveys. We appreciate your commitment’. We used the ‘SurveyImage (SImage)’ tool developed by Höhne, Qureshi, et al. (2020) for taking pictures and uploading images in web surveys (see Appendix C for screenshots). The assignment to treatment groups was constant across all questions. Table 1 summarises the six experimental groups.

**TABLE 1:** Experimental groups

Group name	Device type	Answer format	Motivational message
*PC‐Text*	PC	Text	No
*PC‐Image*	PC	Image	No
*PC‐ImagePush*	PC	Image	Yes
*S‐Text*	Smartphone	Text	No
*S‐Image*	Smartphone	Image	No
*PC‐ImagePush*	Smartphone	Image	Yes

This experiment was placed at the end of a survey with several unrelated experiments. Before the experiment, the questionnaire contained a maximum of 86 questions (depending on other experiments and filtering) about a variety of topics, such as politics, work and personality. To better understand the impact of asking for images on our indicators of interest, we asked participants several questions which varied in terms of the topic and the type of tasks to be performed. The number of experimental questions varied between PC and smartphone groups. We included two experimental questions for PC participants and four for smartphone participants. The first two questions, common to all participants regardless of the device used, required them to upload images that had already been saved. The last two questions were only asked to participants in the smartphone groups and required them to take a photo in the moment. This was done because the availability and use of the camera are more common and easier for smartphones than for PCs (Jäckle et al., 2019). The order of the questions was kept constant across all respondents, hence differences between questions might be produced by specific characteristics of the topic or task and/or by the order.

The formulations of the questions were kept as similar as possible across the text and image experimental groups to maximise the comparability of the results. Only slight variations in the questions and instructions were implemented to request either a text entry or an image from the respondents. More specifically, the experimental questions were the following:

1. **Vacation:** This question asked about the favourite place visited during the participant's last vacation. In this question, the burden of writing a detailed answer was expected to be high. Uploading an image might both provide richer information (e.g. specific landmarks, type of vacations, seasons, etc.) and reduce the burden. The English translation for the *Text* groups was the following: ‘Think about your last vacation. Please, describe the favourite place that you visited (e.g. landscape or monument). *Please type in your answer in the open field below*’. Similarly, the one for the *Image* and *ImagePush* groups was: ‘Think about your last vacation. Please, upload a photo that describes the favourite place that you visited (e.g. landscape or monument). *To select a photo, click on the folder icon above*’.
2. **Dish:** In this question, participants were asked about their favourite dish. This question is an example of how images could provide information that participants are not necessarily aware of. For instance, reporting the different elements of a dish, the size of the portion or even the nutritional value of the dish is something that participants are not necessarily aware of but that can be obtained from specific APIs (see Calorie Mama API http://www.caloriemama.ai/api). Moreover, we expected this question to present low levels of sensitivity for most participants. The English translation for the *Text* groups was the following: ‘Now think about your favourite dish. Please, tell us what your favourite dish is. *Please type in your answer in the open field below*’. For the *Image* and *ImagePush* groups, on the other hand: ‘Now think about your favourite dish. Please, upload a photo of your favourite dish. *To select a photo, click on the folder icon above*’.
3. **Location** (smartphone only): This question asked respondents about the location from which they were answering the survey. This third question is similar to the one used by Bosch et al. (2019a), aimed at substituting multiple questions with one image. It allows us to see if our results are in line with those that Bosch et al. (2019a) found in Spain and Mexico for a sample of Millennials. Specifically, the English translation for *Text* was: ‘From where are you answering this survey? Please describe what you see right now. *Please type in your answer in the open field below*’. In the case of the *Image* and *ImagePush* groups, the wording was as follows: ‘From where are you answering this survey? Please take a photo of what you see right now using your smartphone. *To open the camera, click on the camera icon above*’.
4. **Feeling** (smartphone only): For the final question, we asked respondents how they felt while answering the question, which, for images, translated into them being asked to send a picture of their face. Asking for the participant's face might be useful to detect, using specific APIs, whether the stated demographics of the participants are congruent with the ones detected in the image. In addition, this question might be perceived as more sensitive than the others. The English translation for the *Text* groups was the following: ‘Please describe how you feel right now. *Please type in your answer in the open field below*’. Similarly, the one for *Image* and *ImagePush* groups was: ‘Please take a photo of your face representing how you feel right now using your smartphone. *To open the camera, click on the camera icon above*’.

Additionally, the *Image* and *ImagePush* groups received instructions on how to upload an already‐saved image (PC and smartphone) and on how to take a photo in‐the‐moment (smartphone only). The English translations of the instructions are provided in Appendix B. Furthermore, in order to measure the respondents' evaluations of the experimental questions, two evaluation‐specific questions were asked immediately after the experimental questions to all groups:

1. **Like:** ‘How much did you like answering the last *[two/four]* questions?’
2. **Easy:** ‘How easy or difficult was it to answer these *[two/four]* last questions?’

Both questions used a five‐point, end‐verbalised scale, running from ‘not at all liked it’ to ‘liked it very much’ and from ‘very difficult’ to ‘very easy’.

### 3.2 Data collection

Data were collected in Germany from 15th July to 8th August 2019 using the non‐probability online panel by Respondi (http://www.respondi.com). Respondents received financial compensation from Respondi that was proportional to the estimated survey length. Quotas for age and gender were used to guarantee that the whole sample was similar to the German Micro‐census for individuals living in Germany aged 18–70 years, with respect to those variables. The questionnaire was only available in German, excluding non‐German speakers. The objective was to obtain 3000 respondents finishing the survey (1500 for each device type).

In total, 3043 respondents completed the survey up to the end of our experiment: 558 in *PC‐Text*, 459 in *PC‐Image*, 497 in *PC‐ImagePush*, 579 in *S‐Text*, 476 in *S‐Image* and 474 in *S‐ImagePush*. In total, 1.8% dropped out during the experiment (there was no significant difference between those dropping out and those completing the survey experiment, see the Supplementary Online Material [SOM 1]).

### 3.3 Analyses

#### 3.3.1 Operationalisation

To explore the impact of the different experimental conditions on the four aspects of interest (breakoff, noncompliance, completion times and questions' evaluation), first, we operationalised them into variables (computed for each question):

1. **Breakoff:** a dichotomous variable was created (breakoff = 1, did not breakoff = 0). For each question, we considered that participants broke off the survey if they visited the question page of interest (e.g. *Vacation*) but not the following question page (e.g. *Dish*).
2. **Noncompliance:** a dichotomous variable was created (noncompliance = 1, compliance = 0). This variable was only computed for those who did not breakoff. If nothing was typed or no photo/image was uploaded, this was coded as noncompliance.
3. **Completion time:** a continuous variable (in seconds) was computed. For those who answered, the completion time for each question was measured as the time a respondent clicked ‘next’ on the survey page minus the time this respondent entered the survey page. The completion times were collected using the ‘Embedded Client Side Paradata (ECSP)’ tool (Schlosser & Höhne, 2020). To deal with potential outliers, we used the 3‐SD criterion (three‐sigma rule of thumb). Hence, all completion times that were three standard deviations or more from the mean were considered as outliers and removed. To check the robustness of this method, we also applied the same method as Revilla and Ochoa (2015) to deal with outliers. Similar results were found. Results can be checked in the Supplementary Online Material (SOM 2–4) for both methods.
4. **Questions' evaluation:** two dichotomous variables were created using the questions *Like* and *Easy* (see Section 3.1). For both variables, those who selected the answer categories 4 and 5, indicating that respondents liked or found the experimental questions easy, were categorised as 1. Respondents selecting the remaining options were categorised as 0.

#### 3.3.2 Multivariate analyses

To test our hypotheses, we conducted multivariate analyses. We used two different analysis approaches, one for breakoff, noncompliance and completion time (computed for each question separately), and another for the evaluation of the questions (computed for all the questions together).

**Breakoff, noncompliance and completion time.** To better understand the effect of asking for images instead of text on these indicators, individuals were asked to answer different questions. Questions were, hence, clustered within individuals. Considering this, we decided to use multilevel regressions for each of the three indicators (logistic for breakoff and noncompliance; linear for completion time). The lower level of all our models were the questions (*vacation*, *dish*, *location* and *feeling*), and the higher level were the respondents plus the experimental conditions (a three‐level model with the experimental group as the top level yielded virtually identical results and an overall worse fit).

For each indicator, we ran three models. First, a baseline intercept‐only model (Model 0) to set a baseline with which to compare the other models (Hox et al., 2017). Next, Model 1 included dummy variables representing the experimental groups (e.g. *Text*), the device and the different types of questions (e.g. *Vacation*). This allowed for measuring the effect of *Image* and *ImagePush* and controlling for the type of device and question. Also, since PC and smartphone groups were not equivalent in terms of age, gender and education, and nonresponse slightly unbalanced the subsamples on later questions (see Appendix D), we included these variables as controls. Specifically, age was introduced as a continuous variable and gender and education as dichotomous variables (respectively: men = 0, women = 1; 0 = not completed high education, i.e. post‐secondary education such as university or superior technical training, 1 = completed high education). For Model 2, considering that our experiment followed a 2 × 3 factorial design, and following the advice of Price et al. (2017, pp. 160–167), we additionally included an interaction term between the experimental group and the device. For both Models 1 and 2, we further computed the adjusted predictions at the means (APMs) of each experimental group, to ease interpretation.

After running the regressions, we performed post‐hoc pairwise comparisons (pwcompare in STATA 17) with Bonferroni correction to test the significance of the experimental group's main effects (i.e. the difference between the APMs of *Text*, *Image* and *ImagePush* groups averaged), or the interactions' simple effects (i.e. the differences between subgroup APMs or cells, e.g. *PC‐Image* vs. *S‐Image*). We conducted these post hoc analyses for the model with the highest fit for each indicator, based on the Aikake information criterion (AIC) and the Bayesian information criterion (BIC), as well as the likelihood ratio test ($LR_{X}2$). For indicators in which Model 1 was the one with the highest fit, we assessed the experimental groups' main effects. For indicators in which Model 2 was the one selected, we assessed the simple effects when the interaction was significant and experimental groups' main effects when it was not significant.

**Questions' evaluation.** To analyse the effect of answering with images on the likelihood of participants to like the experimental questions and find them easy, we ran logistic regressions for both questions *Like* and *Easy* separately. For each question we ran two models. Model 1 included dummy variables representing the experimental groups (e.g. *Text*) and the device as well as age, gender and education as controls. Model 2 additionally included an interaction term between the experimental group and the device. For both Models 1 and 2, we further computed the APMs of each variable, to ease interpretation.

After running the regressions, we followed the same approach as for breakoff, noncompliance and question evaluation, performing post hoc pairwise comparisons with Bonferroni correction to test the significance of either the main effects or the simple effects of the models with the highest fit.

## 4 RESULTS

### 4.1 Descriptive results

We first performed some descriptive analyses. Specifically, Table 2 reports the breakoff and noncompliance rates and the average completion time per experimental question, as well as the proportion of participants who liked the experimental questions and found them easy, for each group. For the questions *Vacation, Dish*, *Like* and *Easy,* we also report the marginal means for *Text*, *Image* and *ImagePush* across devices. Marginal means help to better compare the results of asking participants to answer with text or images (with or without a motivational message), regardless of the device used.

**TABLE 2:** Breakoff, noncompliance, completion time and question evaluation by device, and marginal means

	PC			Smartphone			Marginal means		
Indicators	Text	Image	ImagePush	Text	Image	ImagePush	Text	Image	ImagePush
Breakoff (%)									
Vacation	0.0	1.5	0.4	0.0	1.8	1.8	0.0	1.7	1.3
Dish	0.0	0.0	0.2	0.0	1.4	1.4	0.0	0.7	0.8
Location				.2	.2	1.2			
Feeling				0.0	0.6	0.2			
Noncompliance (%)									
Vacation	1.8	38.9	33.1	2.2	25.1	25.3	2.0	31.8	29.2
Dish	1.6	51.6	44.9	.9	39.1	34.9	1.2	45.2	40.0
Location				.5	35.8	33.3			
Feeling				.3	51.5	44.9			
Completion time (avg. sec.)									
Vacation	45.0	78.2	85.9	42.9	65.5	65.6	43.9	71.0	75.3
Dish	31.1	62.0	76.3	31.5	65.6	64.5	31.3	64.1	70.0
Location				26.2	42.9	41.8			
Feeling				19.6	57.5	51.2			
Question evaluation (%)									
Like	52.7	20.0	16.5	47.3	12.4	12.8	50.0	16.1	14.7
Easy	80.1	54.2	51.2	79.0	45.9	45.7	79.5	49.9	48.5

First, it is worth looking at breakoff and noncompliance rates. While breakoff rates were slightly higher for *Image* groups, asking participants to answer with images did not seem to be associated with higher breakoff rates. Table 2, conversely, shows that noncompliance rates for the *Image* and *ImagePush* groups were higher than those for *Text* groups, with differences ranging from 22.9 (*S‐ImagePush*, *Vacation*) to 51.2 (*S‐Image*, *Feeling*) percentage points. Hence, a substantially higher proportion of participants did not comply when asked to answer with images compared to those asked to answer with text.

These results differ from those found by Struminskaya, Lugtig, et al. (2021), who asked participants to send images of their receipts, their house and themselves in a representative general population survey in the Netherlands. While our results show that *Feeling* presents the lowest compliance rate for all questions, Struminskaya and colleagues found that among all the questions tested, the one that yielded the lowest rates was asking for a picture of the participants' house. Moreover, they found a compliance rate of 14.5% for the photo of participants, which is substantially lower than the compliance rate of 48.5% for *S‐Image* and 55.1% for *S‐ImagePush* that our results show. This difference, nonetheless, could be attributed to the fact that (1) both samples are not comparable (Germany vs. the Netherlands; opt‐in online panel vs. probability based), and (2) images were only asked for those who first stated they willingness to comply. Conversely, *Location* is comparable with one of the questions included in Bosch et al. (2019a) (see Section 3.1), who found that between 33% (Mexico) and 45% (Spain) of participants of an opt‐in panel survey uploaded an image. With a noncompliance rate of 36.4% for *S‐Image* and 33.4% for *S‐ImagePush*, the results of this study (Germany) are between those of Bosch et al. (2019a) for a sample of Millennials in Mexico and Spain.

Regarding completion times, *Image* and *ImagePush* groups present longer average completion times (between 15.6 and 45.2 s longer) than *Text* groups. Nonetheless, differences seem to be higher for PC than for smartphone groups. Finally, in terms of question evaluation, 12.4% to 20.0% of the respondents liked answering with images versus 47.3% to 52.7% for the *Text* groups. Moreover, about half of the respondents found the experimental questions easy in the *Image* and *ImagePush* groups, compared to about 80% in the *Text* groups. Overall, these results suggest that only a small to moderate proportion of participants had a positive experience when answering the experimental questions with images.

### 4.2 Multivariate analyses

**FIGURE 1:**
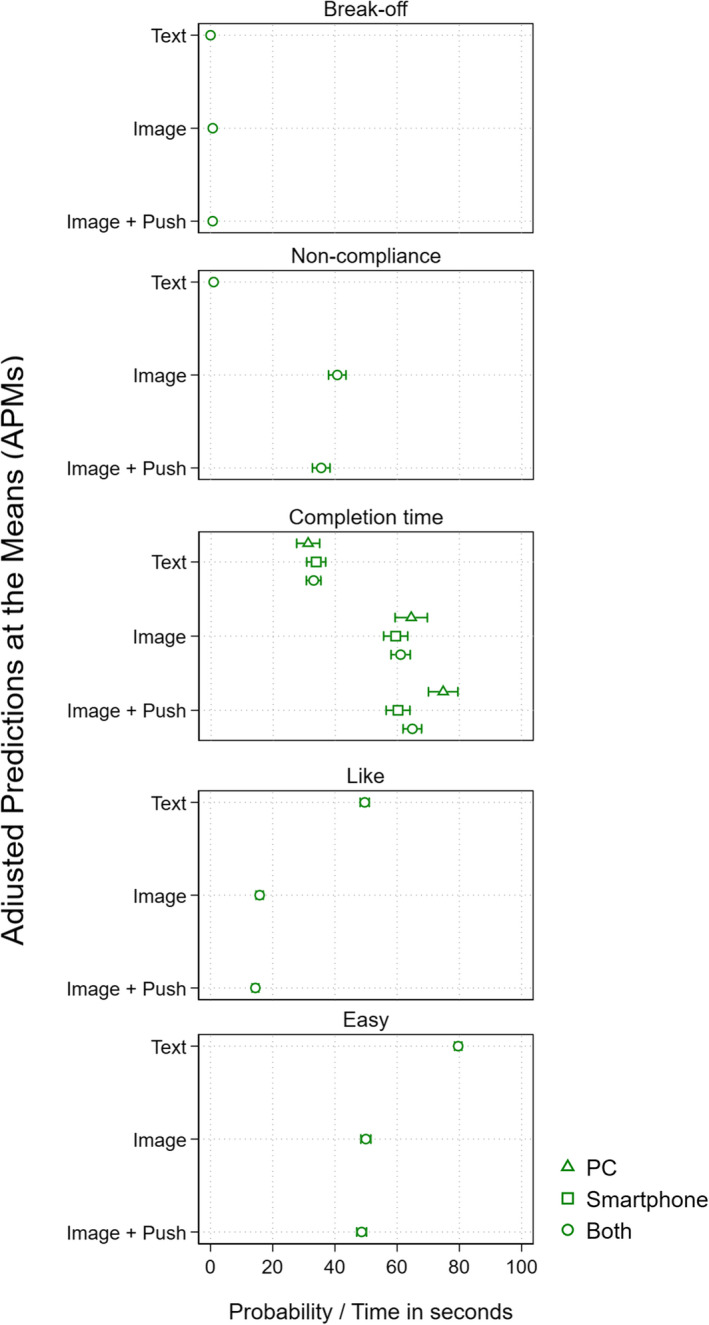
Adjusted predictions at means (APMs). *Note*: APMs reported in probabilities (0% to 100%) for breakoff, noncompliance and *Like* and *Easy*, and in seconds for completion times. Confidence intervals at 95% computed using Bonferroni adjustments.

In order to test our hypotheses, we conducted several multivariate analyses. Table 3 presents the results for the multilevel regression models conducted for breakoff, noncompliance and completion times, while Table 4 shows the results of the logistic regressions conducted for *Like* and *Easy*. In addition, Figure 1 shows all the APMs for the experimental groups, allowing to visually check the size of the differences between groups (e.g. *Image* vs. *ImagePush*) and subgroups (*PC‐Image* vs. *S‐Image*). The significance levels of the relevant pairwise comparisons are presented in the text and can be consulted in more detail in the Supplementary Online Material (SOM 5).

#### 4.2.1 Breakoff

First of all, Model 0 presents an interclass correlation (ICC) of 0, meaning that 0% of the variance in the likelihood of breaking‐off is explained by the clustering of the observations (questions) within individuals and experimental groups. Therefore, breakoff is a characteristic of the questions, not the individual or the experimental question. This is to be expected considering the small incidence of breakoffs across all experimental groups. This low incidence and variation are also reflected in both Models 1 and 2; no significant effect for the experimental groups nor for the interaction terms was observed. Looking at the predicted probabilities (APMs) of abandoning the survey for Model 1 (best fit), we observed that *Image* and *ImagePush* significantly increased these by 0.70 and 0.68 percentage points compared to *Text* (both *ρ* = 0). In addition, *Image* and *ImagePush* differed by a non‐significant 0.02 percentage point (*ρ* = 1.00). Regardless of the significance of these differences, one must consider that probabilities were small across groups and questions (see APMs in both Table 3 and Figure 1) and the effect sizes of these were small as well. Hence, the results seem to mostly go against hypotheses *H1a*, *H2a* and *H3a*: there are no relevant differences across groups, and these do not depend on the type of device.

**TABLE 3:** Multilevel regression coefficients and adjusted predictions at the means (APMs) for breakoff, noncompliance and completion time

	Breakoff				Noncompliance				Completion time			
	Model 1		Model 2		Model 1		Model 2		Model 1		Model 2	
Fixed effects	Coeff.	APM	Coeff.	APM	Coeff.	APM	Coeff.	APM	Coeff.	APM	Coeff.	APM
Intercept	−9.58				−8.91**		−8.91**		31.6**		27.6**	
	(1.2)				(0.57)		(0.66)		(3.55)		(3.72)	
*Group*												
Text (ref.)		0.02		0.00		0.97		0.96		32.36		33.04
		(0.02)		(0.00)		(0.23)		(0.23)		(1.19)		(1.19)
Image	3.47	0.72	4.25	0.77	7.97**	40.71	7.97**	40.58	28.77**	61.13	33.22**	61.08
	(1.01)	(0.16)	(1.23)	(0.16)	(0.43)	(1.43)	(0.58)	(1.44)	(1.97)	(1.58)	(3.16)	(1.58)
ImagePush	3.45	0.71	3.34	0.71	7.35**	35.54	7.34**	35.73	33.41**	65.77	43.52**	64.86
	(1.02)	(0.15)	(1.02)	(0.16)	(0.42)	(1.44)	(0.56)	(1.47)	(1.91)	(1.51)	(2.97)	(1.52)
Smartphone	1.16		1.76		−0.99		−1.12		−4.23*		2.59	
	(0.38)		(0.63)		(0.2)		(0.67)		(1.81)		(2.52)	
*Question*												
Dish	−0.6		−0.6		1.13**		1.13**		−9.36**		−9.3**	
	(0.32)		(0.32)		(0.11)		(0.11)		(1.24)		(1.24)	
Location	−0.96		−0.96		0.88**		0.88**		−20.83**		−20.87**	
	(0.41)		(0.41)		(0.14)		(0.14)		(1.61)		(1.61)	
Feeling	−1.65		−1.65		2.1**		2.1**		−19.45**		−19.6**	
	(0.54)		(0.54)		(0.14)		(0.14)		(1.67)		(1.67)	
*Resp. characteristics*												
Women	0.38		0.38		−0.14		−0.14		−1.36		−1.52	
	(0.29)		(0.29)		(0.19)		(0.19)		(1.66)		(1.65)	
Age	0.02		0.02		0.00		0.00		0.31**		0.31**	
	(0.01)		(0.01)		(0.01)		(0.01)		(0.06)		(0.06)	
Education	0.12		0.13		−0.31		−0.31		−0.47		−0.38	
	(0.29)		(0.29)		(0.19)		(0.19)		(3.55)		(1.66)	
*Interaction*												
Text_PC (ref.)				0				1.59				31.27
				(0.00)				(0.48)				(1.89)
Text_S (ref.)				0.04				0.74				33.67
				(0.04)				(0.25)				(1.56)
Image_PC (ref.)				0.46				48.02				64.5
				(0.19)				(2.12)				(2.65)
ImagePush_PC (ref.)				0.19				41.75				74.79
				(0.11)				(2.12)				(1.98)
Image_S			−1.09	0.9			0.02	36.79			−7.61	59.49
			(0.76)	(0.21)			(0.77)	(1.93)			(4.03)	(2.42)
ImagePush_S			0.00	1.07			0.26	32.77			−17.15**	60.24
			(0.00)	(0.23)			(0.72)	(1.92)			(3.86)	(1.96)
*Random effects*												
Second‐level variance	0.00		0.00		11.00		10.99		922.7		907.74	
	(0.00)		(0.00)		(0.99)		(0.92)		(52.39)		(52.01)	
First‐level variance	3.29		3.29		3.29		3.29		1742.38		1743.65	
									(38.81)		(38.85)	
ICC	0.00		0.00		0.77		0.77		0.35		0.34	
*Model fit*												
Loglikelihood	−299.76		−298.43		−3218.85		−3218.61		−36,755		−36,745.82	
LR × 2 to prev. model	65.88**		NA${\;}^{2}$		1398.26**		0.47		671.07**		19.69**	
AIC	619.51		618.87		6459.7		6463.23		73,535.32		73,519.63	
BIC	691.04		696.13		6538.1		6555.88		73,617.43		73,615.43	
*N respondents*	3131		2558		3079		3079		2597		2597	
*N observations*	9442		8296		9203		9203		6922		6922	

*Note*: Coefficients are presented in log odds for breakoff and noncompliance rates (logistic model), and in seconds for completion times (linear model). APMs presented for the experimental groups and their interactions with device type, regardless of whether they are reference groups or not. The random effects and the model fit values for the baseline models (Model 0) for each of the indicators are the following (order: breakoff, noncompliance and completion times): Respondent variance = 0.00 , 15.41, 1243.81; ICC = 0.00, .82, .41; Loglikelihood = −332.70, −3917.98, −37,091.20; AIC = 667.40, 7839.96, 74,188.39; BIC = 674.55, 7854.21, 74,208.92. (1) For the logistic models the lower‐level variance is fixed at $\left(\frac{\pi^{2}}{3}\right)$ (Hox et al., 2017, p. 117). (2) Different number of observations for each model, due to the exclusion of participants from subgroups with constant values (i.e. no breakoffs happened in *TextPC*). *p < 0.05; **p < 0.01.

**TABLE 4:** Logistic regression coefficients and APMs for *Like* and *Easy*

	Like				Easy			
	Model 1		Model 2		Model 1		Model 2	
	Coeff.	APM	Coeff.	APM	Coeff.	APM	Coeff.	APM
*Intercept*	−0.18		−0.23		1.05**		0.96**	
	(0.19)		(0.19)		(0.17)		(0.19)	
*Group*								
Text (ref.)		49.58		49.56		79.62		79.56
		(1.5)		(1.5)		(1.12)		(1.2)
Image	−1.66**	15.8	−1.51**	15.59	−1.37**	49.94	−1.22**	49.96
	(0.11)	(1.2)	(0.15)	(1.21)	(0.99)	(1.64)	(0.14)	(1.65)
ImagePush	−1.76**	14.44	−1.73**	14.47	−1.42**	48.58	−1.34**	48.58
	(0.11)	(1.13)	(0.15)	(1.14)	(0.1)	(1.61)	(0.14)	(1.61)
Smartphone	−0.28**		−0.19		−0.2		−0.04	
	(0.09)		(0.12)		(0.08)		(0.15)	
*Resp. characteristics*								
Women	0.13		0.13		0.1		0.11	
	(0.09)		(0.09)		(0.08)		(0.08)	
Age	0.01**		0.01**		0.01**		0.01	
	(0.00)		(0.00)		(0.00)		(0.00)	
Education	−0.33**		−0.32**		0.01		0.02	
	(0.09)		(0.09)		(0.08)		(0.08)	
*Interaction*								
Text_PC (ref.)				52.01				79.9
				(2.18)				(1.71)
Text_S (ref.)				47.14				79.22
				(2.13)				(1.71)
Image_PC (ref.)				19.37				53.95
				(1.86)				(2.37)
ImagePush_PC (ref.)				16.08				51.06
				(1.65)				(2.28)
Image_S			−0.33	12.47			−0.28	46.03
			(0.22)	(1.53)			(0.2)	(2.32)
ImagePush_S			0.05	13.01			−0.16	46.14
			(0.22)	(1.57)			(0.2)	(2.32)
*Model fit*								
Loglikelihood	−1581.07		−1579.91		−1882.25		−1881.28	
LR × 2 to prev. model			2.32				1.94	
AIC	3176.14		3177.82		3778.5		3780.56	
BIC	3218.27		3231.99		3820.63		3834.72	
*N respondents*	3039		3039		3034		3034	

*Note*: Coefficients are presented in log odds. APMs presented for the experimental groups and their interactions with device type, regardless of whether they are reference groups or not. *p < 0.05; **p < 0.01.

#### 4.2.2 Noncompliance with the task

Regarding noncompliance, Table 3 shows that the ICC for Model 0 was 0.82, indicating that 82% of the variance in the likelihood of noncompliance is explained by the clustering of the observations. Noncompliance is, hence, mostly a characteristic of the individual and the experimental condition, rather than the question. In terms of Model 1, which presents the best fit, it was observed that the likelihood of noncompliance significantly differed between *Text*, *Image* and *ImagePush*. Specifically, while the predicted probability of noncompliance (APMs in Table 3 and Figure 1) for individuals in the *Text* group was 0.97%, for those in *Image* and *ImagePush* it was 40.71% and 35.54%, respectively. Asking for images, consequently, increased the probability of noncompliance by around 34%–39% points (both differences at *ρ* = 0.00). These results strongly support *H1b*. Focusing on the effect of motivational messages and comparing the APMs of *Image* and *ImagePush* from Table 3 and Figure 1, we see the latter presenting a 5.17% point significantly lower predicted probability of noncompliance (*ρ* = 0.029). Hence, in line with *H2b*, the results imply that adding a motivational message does increase the participant's propensity to comply.

Although Model 2 did not improve the overall fit compared to Model 1, its results show that no interaction terms are significant, contradicting *H3b*. Moreover, the APMs for the main effects of *Text*, *Image* and *ImagePush* were virtually the same as for Model 1.

#### 4.2.3 Completion times

Moving to completion times, first, results from Model 0 showed an ICC of 0.41, indicating that 41% of the variance in the likelihood of breaking‐off is explained by the clustering of the observations; completion times were mostly a characteristic of the question rather than the individual and the experimental condition. Although Models 1 and 2 show similar ICC values, Model 2—the one yielding the best fit—also suggests that asking for an image instead of text has a significant impact on completion times and that there is an interaction between this impact and the device used to participate. Specifically, results show that *Image* and *ImagePush* groups present significantly higher predicted completion times (APMs) than *Text* groups, between 25.62 (*S‐Text* vs. *S‐Image, ρ* = 0.000) and 43.52 (*PC‐Text* vs. *PC‐ImagePush, ρ* = 0.000) more. Hence, participants systematically take more time answering with images than with text, regardless of the device, supporting *H1c*.

The results also show evidence that the effect of asking participants to answer with an image is lower for smartphone than PC participants, but only for those prompted with a motivational message. Regarding the predicted completion times (APMs), while no significant difference was observed between *PC‐Image* and *S‐Image* (*ρ* = 1.000), *PC‐ImagePush* presented completion times that were 14.55 s longer than *S‐ImagePush* (*ρ* = 0.000). Even if these results are not contrary to *H3c*, the evidence is still conflicting, since the type of device only moderates the increase in completion times of asking participants to answer with images for those prompted with a motivational message. This interaction between device and the use of motivational messages can also be observed when looking at the difference between *Image* and *ImagePush* across devices. While adding a motivational message does not influence the completion times for smartphone participants (*ρ* = 1.000), we see an increase of 10.3 seconds for those answering with images on a PC, which falls within the threshold of significance (*ρ* = 0.050, this difference is not significant in the 99% Model, see the Supplementary Online Material (SOM 4)). In other words, although adding a motivational message does not affect completion times for participants answering with a smartphone, it might increase the times when answering on a PC. Again, evidence in favour of *H2c* is dependent on the devices used.

#### 4.2.4 Questions' evaluation

Finally, regarding question evaluation, Model 1 (best fit) shows that the likelihood of both liking the questions and finding them easy is significantly higher for *Text* than *Image* and *ImagePush*. Specifically, compared to *Text*, *Image* and *ImagePush* participants present significantly lower predicted probabilities (APMs) of liking the questions (49.58% vs. 15.80% and 14.44% respectively, *ρ* = 0.000 for both) and finding them easy (79.62% vs. 49.94% and 48.58% respectively *ρ* = 0.000 for both). This is in line with *H1d*. Conversely, the small differences observed between *Image* and *ImagePush* are not significant for both *Like* (*ρ* = 0.290) and *Easy* (*ρ* = 0.710), meaning that adding a motivational message does not significantly affect the participants' propensity of liking the questions and finding them easy. This goes in favour of *H2d*.

However, while Model 2 does not improve the overall fit compared to Model 1, on further inspection, it shows that none of the interaction terms is significant, contradicting *H3d*. Moreover, the APMs for *Text*, *Image* and *ImagePush* are virtually the same as for Model 1.

## 5 DISCUSSION AND CONCLUSIONS

### 5.1 Main results

In this study, we used data from the Respondi opt‐in online panel in Germany to address three research questions on the impact of being asked to answer through images instead of typing text (**RQ1**), of presenting a motivational message (**RQ2**), and on whether there is an interaction between asking for images and the device used (**RQ3**), on four aspects: breakoff, noncompliance, completion time and the extent to which participants liked the experimental questions and found them easy.

Overall, we found support for five hypotheses:

Asking participants to answer with images compared to asking them to answer with text led to a strong increase in the participants' likelihood of noncompliance (*H1b*) and their completion times (*H1c*), as well as to a substantial reduction in the likelihood of them answering the experimental questions and liking them, and a reduction in them finding the questions easy to answer (*H1d*). *Including a motivational message when asking to answer with an image reduced the participants' likelihood of noncompliance (H2b) to some extent, while not affecting their likelihood of liking the experimental questions and finding them easy (H2d)*.

For the other hypotheses, we either found mixed or no supporting evidence. The implications of these results are further discussed in the following subsections.

#### 5.1.1 Impact of answering with images instead of text (RQ1)

Asking participants to answer with images instead of text does not increase breakoff rates, but it does substantially increase noncompliance rates. Although we did not explore the reasons behind this lower likelihood of complying with the tasks, it could be linked to the fact that asking for images also significantly increases the time spent to comply with the tasks. If participants take more time not because they put more thought and care into the task, but because the task is in itself more burdensome and complex, this could deter participants from complying. Indeed, our results show that asking participants to answer with images leads to a more negative survey experience, with participants showing a higher likelihood of saying that they did not like the experimental questions nor found them easy. This goes against the idea that answering with images could increase participants' engagement because it would make the survey experience more natural for them (Bosch et al., 2019a; Link et al., 2014), aligning with the explanation that answering with images might be more burdensome and challenging.

Although other reasons can still justify asking for images in the context of a survey, researchers must consider the potential effect of doing so on noncompliance rates when assessing whether and when asking participants to answer with images could be useful.

#### 5.1.2 Effect of including a motivational message (RQ2)

We found that adding motivational messages when asking participants to answer with images increases the likelihood of participants complying with the tasks. We additionally found that adding a motivational message might increase completion times for participants answering through a PC, but not for those using smartphones. Whether this happened because participants using PCs put more care into the answering than those not being prompted by a motivational message or using a smartphone, or because of other explanations, remains unclear.

These results are in line with previous research, which has generally found that motivational messages either lead to insignificant effects or rather small significant ones (Bosch et al., 2018; Kapelner & Chandler, 2010; Sakshaug & Crawford, 2010). Regardless of the size of the effect, considering the inexpensive nature of motivational messages and their low tendency of introducing negative effects, it seems recommendable to introduce them when asking for images given their potential to reduce noncompliance rates.

#### 5.1.3 Interaction with the device type (RQ3)

For breakoff, noncompliance and question evaluations the impact of being asked to answer with an image (with or without motivational message) did not significantly differ between devices, whereas for completion times it did. Nonetheless, this interaction was only present for *ImagePush* which, as discussed in the previous subsection, suggests that the difference in slopes is associated with motivational messages affecting differently those individuals participating through PCs than those using smartphones.

Even if individuals are less familiar with the procedure of uploading images from their PCs than from their smartphones (Carrington, 2020; Mendelson & Papacharissi, 2010), our results suggest that no differences between devices should be expected in terms of the impact of asking for images compared to asking participants to answer with text on the four indicators.

### 5.2 Limitations and future research

Our study has some limitations that future research could address. First, we used data from an online opt‐in panel. Although quotas were applied to represent the general German population in terms of age and gender, opt‐in panellists are more accustomed to answering surveys than the general population. Thus, their ability and willingness to upload images may differ from the general population. Second, our PC and smartphone subsamples were unbalanced in terms of age and gender. Although the analyses examining the interaction between devices controlled for these sociodemographic variables, this procedure is not equivalent to using the device as a quota variable. Moreover, we did not look at data quality indicators as the information conveyed or the validity of the answers. Furthermore, since we could not ask respondents to stop the survey and answer at a later point in time, we had to request photos that respondents could take in the moment. Thus, the images we could request were limited. For instance, we could not ask respondents to take a photo of their fridge to study their nutrition behaviour, since many respondents might not be answering the survey from home. Finally, since the order of the questions was not randomised, differences between questions could be due to this and not to intrinsic differences between topics. Hence our decision to not explore interactions between question topics, experimental groups and devices.

Further research should try to overcome these limitations and test the robustness of the results for different settings. Beyond these limits, much research is still needed to better understand the impact of asking participants to answer survey questions with images and under which circumstances this is recommendable. Specifically, research trying to identify the potential benefits of asking for images is still lacking. Future research could explore whether images can increase the quality of measurements and whether there are cases in which asking for images can reduce the participants' burden.

In addition, although the motivational messages were effective, more research should explore complementary strategies to decrease noncompliance. Specifically, uploading images is not only related to participant willingness, but also to other factors such as whether they understand how to perform the required task (Bosch et al., 2019a, 2019b) or if they are in a situation to perform the task (e.g. they cannot take a picture of their fridge if they are not at home). Therefore, apart from exploring alternative strategies to increase the willingness to participate, specific strategies for other potential limitations should be explored (e.g. providing targeted instructions, asking only when we know that they are in a situation of complying, etc.).

Finally, although we found no interaction between asking for images and the device type, our findings might not be generalisable to other tasks. Beyond the studied indicators, further research should also study whether the effect of asking for images on measurement quality indicators (e.g. number of topics, richness, etc.) is moderated by the device type, given that we expect PCs to have a more limited availability of visual data compared to smartphones (this has been indeed found by Iglesias and Revilla (2021)).

### 5.3 Conclusions

This research demonstrates that using images to answer online survey questions still present many challenges, the main one being the substantial increase of the likelihood of noncompliance with the tasks, compared with the more traditional approach of asking for written text. Although we found that this likelihood can be reduced by prompting participants with motivational messages, noncompliance rates of 25.1%–51.5% can still pose challenges to those wanting to ask for images instead of text. Nonetheless, if these negative consequences are partially due to a higher burden of answering with images, as the higher completion times could suggest, the more participants are used to share images within the frame of surveys, the lower the negative consequences should be over time. However, this is yet to be tested.

Despite the negative results, asking for images might still be an overall positive approach for those topics with which images can improve the information that can be obtained or in those cases in which traditional surveys cannot collect the desired information. For instance, recent research has shown that asking for screenshots of smartphone screen‐time information (e.g. iOS Screen Time) allows for the collection of higher quality data since self‐reports are significantly biased, at the cost of introducing some rather marginal nonresponse biases (Ohme et al., 2020). These trade‐offs between representation and measurement errors must be better explored for different topics, to understand when images should or should not be used. As Ohme et al. (2020)'s example shows, although representation errors could be higher when asking to share images (e.g. in terms of mobile privacy literacy), measurement errors can potentially be reduced to an extent to which the overall data quality improves. Thus, when deciding whether to ask for images or not, researchers should consider the balance between both error dimensions. Beyond this, there might be cases in which asking for images could reduce the participants' burden to answer. For instance, in cases like batteries, diaries or long and detailed open‐ended questions, images have the potential to substitute more than one question and/or complex and burdensome tasks. In these cases, although our results seem to indicate that asking for images increases participant burden, sharing one image could indeed be a less burdensome option compared to traditional survey questions (Link et al., 2014). There is, indeed, some evidence showing that individuals perceive sharing an image as less burdensome than answering survey batteries (Iglesias & Revilla, 2021).

Overall, although these results caution researchers about the potential problems of asking participants to answer with images, it also shows that it is overall feasible to do so and obtain compliance rates above 40% in certain populations (keeping in mind the non‐probability nature of the panel). However, more refined methodological research is needed to determine when and how images can meaningfully substitute or be combined with conventional survey questions.

## APPENDIX A ENGLISH TRANSLATIONS OF THE INSTRUCTIONS

### A.1 For uploading an already saved image

Next, we will ask you to answer some questions uploading images from your [PC/smartphone].

First, click on the folder icon. This will open your [PC folders/ smartphone gallery]. Search for a photo in your [PC folders/ smartphone gallery] as you normally do. Once selected an image from your [PC folders/ smartphone gallery], a miniature of the image will appear instead of the folder icon.

If you are satisfied with the image that appears, click the ‘continue’ button to get to the next question. If you want to change the image, you can click the delete button.

Then, you will be able to search for a new image following the same process. You can repeat this process until you are satisfied with the image. Then, click ‘Continue’.

### A.2 For taking a photo in‐the‐moment (smartphone only)

Next, we will ask you to answer some questions taking photos with your smartphone.

First, click on the camera icon. This will open the camera of your smartphone. Take the photo as usually. A miniature of the photo will appear instead of the camera icon.

If you are satisfied with the photo that appears, click the ‘continue’ button to get to the next question. If you want to change the photo, you can click the delete button.

Then, you will be able to take a new photo following the same process. You can repeat this process until you are satisfied with the photo. Then, click ‘Continue’.

## APPENDIX B SCREENSHOTS OF THE TOOL FOR UPLOADING AN IMAGE AND TAKING A PHOTO IN THE MOMENT

*Note*: The PC version for uploading an image is presented on the left side (*Vacation*). The smartphone version for uploading an image is presented in the middle (*Vacation*). The smartphone version for taking a photo in‐the‐moment is presented on the right side (*Location*).

## APPENDIX C SAMPLE COMPOSITION OF THE EXPERIMENTAL GROUPS

**Table:**

	PC			Smartphone		
	*Text*	*Image*	*ImagePush*	*Text*	*Image*	*ImagePush*
Female (%)	**37.0**	**34.5**	**36.7**	65.4	63.2	60.4
Age (mean)	**50.3**	**50.2**	**49.2**	43.5	43.9	42.7
Highly educated (%)	49.2	**51.6**	51.7	49.0	58.2**	57.6**

*Note*: **p* < 0.05; ***p* < 0.01. Asterisks in the Image and *ImagePush* columns indicate significant differences between the text and image groups. **Bold** in PC columns indicates a significant difference between devices within a given group.

## APPENDIX D Sample composition of the experimental groups, excluding non‐respondents

**Table:**

	PC			Smartphone		
	*Text*	*Image*	*ImagePush*	*Text*	*Image*	*ImagePush*
***Travel***						
Female (%)	**37.6**	**32.7**	**37.7**	65.8	62.5	60.5
Age (mean)	**50.6**	**48.6**	**49.8**	43.6	45.2	42.4†
Highly educated (%)	49.1	54.8	53.3	48.9	59.8**	57.2*
***Dish***						
Female (%)	**37.5**	**32.0**	**38.3**	65.6	61.2	62.5
Age (mean)	**50.6**	45.8**	**48.3***	43.5	44.1	41.5†
Highly educated (%)	49.2	56.8	55.8	48.9	60.9**	57.8*
***Location***						
Female (%)				65.3	61.4	61.2
Age (mean)				43.4	45.4	41.7†
Highly educated (%)				49.0	58.2**	56.8*
***Feeling***						
Female (%)				65.3	59.3	59.0
Age (mean)				43.4	46.2*	41.9†
Highly educated (%)				49.0	62.3**	54.0

*Note*: **p* < 0.05; ***p* < 0.01. Asterisks in the *Image* and *ImagePush* columns indicate significant differences between the text and image groups. **Bold** in PC columns indicates a significant difference between devices within a given group. ^†^in *ImagePush* columns indicates a significant difference between the Image and *ImagePush* groups.
