# Supplementary material for: A new experiment on the use of images to answer web survey questions
Source: J R Stat Soc Ser A Stat Soc. 2022 May 20;185(3):955–80. doi: 10.1111/rssa.12856 (PMC9543211; doi:10.1111/rssa.12856)
Supplement: Supplementary file 1 [file RSSA-185-955-s001.docx]

**Supplementary Online Material (SOM)**

SOM1 presents the proportions and averages for the main sociodemographic used in the paper. Results are presented for each sub-group (e.g. PC-Text) and, within those groups, for those individuals that abandoned the survey at some point of the experiment and those that stayed. The significance of the differences were analysed with t-test (for averages) or Z-tests (for proportions).

**SOM1.** Differences between those that brokeoff during the experiment and those who stayed in terms of sociodemographics.

|  | *PC* | | | | | | *Smartphone* | | | | | |
| --- | --- | --- | --- | --- | --- | --- | --- | --- | --- | --- | --- | --- |
|  | *Text* | | *Image* | | *ImagePush* | | *Text* | | *Image* | | *ImagePush* | |
|  | *Stayed* | *Broke-off* | *Stayed* | *Broke-off* | *Stayed* | *Broke-off* | *Stayed* | *Broke-off* | *Stayed* | *Broke-off* | *Stayed* | *Broke-off* |
| Female (%) | 37.0 | NA | 34.1 | 57.1 | 36.7 | 33.3 | 65.4 | 100 | 63.1 | 65 | 60.0 | 69.6 |
| Age (mean) | 50.3 | NA | 50.0 | 59.9 | 49.1 | 58 | 43.4 | 56.0 | 43.8 | 44.5 | 42.6 | 45.6 |
| Highly educated (%) | 49.2 | NA | 51.3 | 71.4 | 51.8 | 33.3 | 49.0 | 0.0 | 58.3 | 55.0 | 57.7 | 56.5 |
| n | 573 | 0 | 460 | 7 | 498 | 3 | 589 | 1 | 480 | 20 | 477 | 23 |

*Note*: no significant difference between *Broke-off* and *Stayed* groups.

SOM2 presents the average completion times for each question and sub-group. Completion times are presented for two different approaches to deal with outliers: the 3-SD criterion, used in the main document, and the 99% approach which has been used in other papers dealing with similar problems (e.g. Revilla and Ochoa, 2015; Bosch and Revilla, 2020). For this approach we replaced the values for the 1% respondents with the highest completion time by the average completion time of the remaining 99% (after excluding non-respondents). We used the average time and not the maximum time of the other 99% because, following Revilla and Ochoa (2015), we believe that very long completion times do not only indicate extremely slow respondents but respondents who interrupted the survey.

**SOM2.** Average completion times per experimental group, for both approaches to deal with outliers.

|  | **PC** | | | **Smartphone** | | | **Marginal means** | | |
| --- | --- | --- | --- | --- | --- | --- | --- | --- | --- |
|  | *Text* | *Image* | *ImagePush* | *Text* | *Image* | *ImagePush* | *Text* | *Image* | *ImagePush* |
| 3-SD (*in sec.*) |  |  |  |  |  |  |  |  |  |
| *Vacation* | 45.0 | 78.2 | 85.9 | 42.9 | 65.5 | 65.6 | 43.9 | 71.0 | 75.3 |
| *Dish* | 31.1 | 62.0 | 76.3 | 31.5 | 65.6 | 64.5 | 31.3 | 64.1 | 70.0 |
| *Location* |  |  |  | 26.2 | 42.9 | 41.8 |  |  |  |
| *Feeling* |  |  |  | 19.6 | 57.5 | 51.2 |  |  |  |
| 99% (*in second*) |  |  |  |  |  |  |  |  |  |
| *Vacation* | 42.1 | 76.5 | 80.4 | 42.3 | 64.1 | 63.1 | 42.3 | 69.5 | 71.4 |
| *Dish* | 30.0 | 59.0 | 67.9 | 30.5 | 61.6 | 57.8 | 30.3 | 60.5 | 62.5 |
| *Location* |  |  |  | 27.9 | 43.7 | 42.1 |  |  |  |
| *Feeling* |  |  |  | 18.8 | 50.0 | 46.3 |  |  |  |

SOM3 presents the results of the multilevel regressions for both approaches to deal with outliers.

**SOM3.** Multilevel regression coefficients and APMs for completion times, for both approaches to deal with outliers.

|  | ***3-SD*** | | | | ***99%*** | | | |
| --- | --- | --- | --- | --- | --- | --- | --- | --- |
|  | ***Model 1*** | | ***Model 2*** | | ***Model 1*** | | ***Model 2*** | |
| Fixed effects | ***Coeff.*** | ***APM*** | ***Coeff*** | ***APM*** | ***Coeff.*** | ***APM*** | ***Coeff.*** | ***APM*** |
| *Intercept* | 31.60**  (3.55) |  | 27.60**  (3.72) |  | 30.75**  (2.76) |  | 26.51**  (2.88) |  |
| *Group* |  |  |  |  |  |  |  |  |
| *Text (ref.)* |  | 32.36  (1.19) |  | 33.04  (1.19) |  | 31.46  (.92) |  | 32.15  (.93) |
| *Image* | 28.77**  (1.97) | 61.13  (1.58) | 33.22**  (3.16) | 61.08  (1.58) | 27.14**  (1.53) | 58.60  (1.23) | 33.01**  (2.46) | 58.44  (1.22) |
| *ImagePush* | 33.41**  (1.91) | 65.77  (1.51) | 43.52**  (2.97) | 64.86  (1.52) | 29.34**  (1.49) | 60.80  (1.18) | 38.44**  (2.31) | 60.06  (1.18) |
| *Device* |  |  |  |  |  |  |  |  |
| *PC (ref.)* |  |  |  |  |  |  |  |  |
| Smartphone | -4.23*  (1.81) |  | 2.59  (2.52) |  | -3.38*  (1.41) |  | 3.53  (1.96) |  |
| *Question* |  |  |  |  |  |  |  |  |
| Vacation (ref.) |  |  |  |  |  |  |  |  |
| Dish | -9.36**  (1.24) |  | -9.30**  (1.24) |  | -10.31**  (.97) |  | -10.25**  (.97) |  |
| Location | -20.83**  (1.61) |  | -20.87**  (1.61) |  | -18.59**  (1.26) |  | -18.62**  (1.26) |  |
| Feeling | -19.45**  (1.67) |  | -19.60**  (1.67) |  | -20.73**  (1.31) |  | -20.89**  (1.31) |  |
| Resp. characteristics |  |  |  |  |  |  |  |  |
| Women | -1.36  (1.66) |  | -1.52  (1.65) |  | -.87  (1.29) |  | -.99  (1.28) |  |
| Age | .31**  (.06) |  | .31**  (.06) |  | .29**  (.04) |  | .30**  (.04) |  |
| Education | -.47  (3.55) |  | -.38  (1.66) |  | -.06  (1.29) |  | -.07  (1.29) |  |
| *Interaction* |  |  |  |  |  |  |  |  |
| *Text_PC (ref.)* |  |  |  | 31.27  (1.89) |  |  |  | 29.74  (1.89) |
| *Text_S (ref.)* |  |  |  | 33.67  (1.56) |  |  |  | 33.27  (1.56) |
| *Image_PC (ref.)* |  |  |  | 64.5  (2.65) |  |  |  | 62.75  (2.65) |
| *ImagePush_PC (ref.)* |  |  |  | 74.79  (1.98) |  |  |  | 68.17 (1.88) |
| *Image*_S |  |  | -7.61  (4.03) | 59.49  (2.42) |  |  | -9.84**  (3.13) | 56.45  (1.98) |
| *ImagePush*_S |  |  | -17.15**  (3.86) | 60.24  (1.96) |  |  | -15.42**  (3.00) | 56.29  (1.52) |
| Random effects |  |  |  |  |  |  |  |  |
| *Second-level variance* | 922.70  (52.39) |  | 907.74  (52.01) |  | 553.04  (30.91) |  | 540.66  (30.58) |  |
| *First-level variance* | 1,742.38 (38.81) |  | 1,743.65  (38.85) |  | 1,076 (23.58) |  | 1,077.09  (23.60) |  |
| ICC | .35 |  | .34 |  | .34 |  | .33 |  |
| Model fit |  |  |  |  |  |  |  |  |
| LogLikelihood | -36,755 |  | -36,745.82 |  | -35,277.69 |  | -35,263.63 |  |
| LR x2 to prev. model | 671.07** |  | 19.69** |  | 935** |  | 935** |  |
| AIC | 73,535.32 |  | 73,519.63 |  | 70,579.38 |  | 70,555.25 |  |
| BIC | 73,617.43 |  | 73,615.43 |  | 70,661 |  | 70,651.13 |  |
| *N* respondents | 2,599 |  | 2,5979 |  | 2,599 |  | 2,599 |  |
| *N* observations | 6,964 |  | 6,964 |  | 6,964 |  | 6,964 |  |

*Note.* Coefficients are presented in seconds for completion times. APMs presented for the experimental groups and their interactions with device type, regardless of whether they are reference groups or not. The random effects and the model fit values for the baseline models (model 0) for 3-SD and 99% are the following, respectively: Respondent variance = 1243.81, 1,151.23; ICC = ..41, .41; LogLikelihood = -37,091.20, -35,745.65; AIC = 74,188.39, 71,487.29; BIC 74,208.92, 71,517.84.

*p < .05. **p < .01.

SOM 4 presents the adjusted predictions at the means (APMs) for each question and subgroup, for both approaches to deal with outliers.


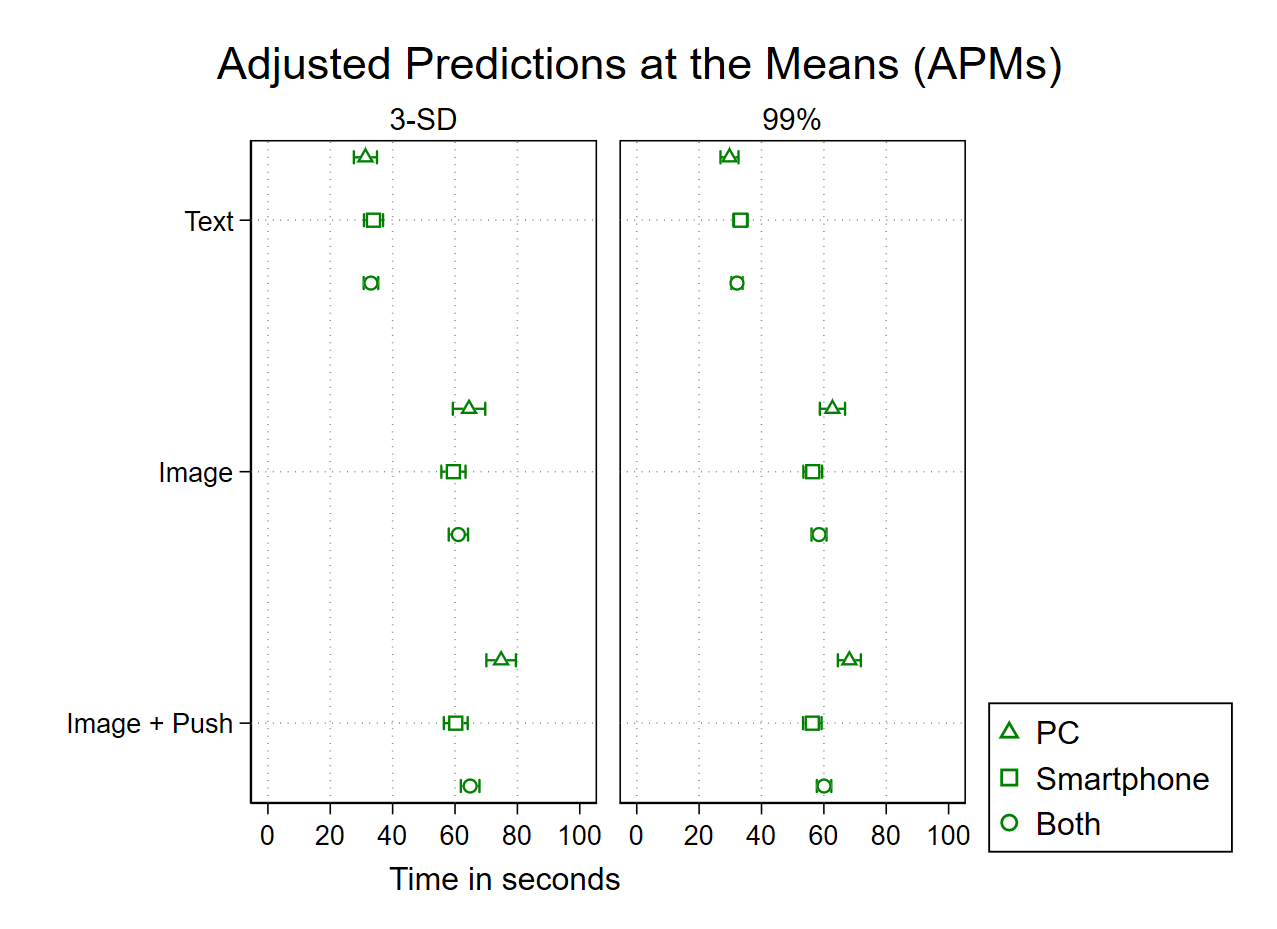


**SOM4**. APMs for both approaches to deal with outliers. *Note:* Adjusted Predictions at Means (APMs) in seconds for completion times. Confidence Intervals at 95% computed using Bonferroni adjustments.

SOM 5 presents the relevant pairwise comparisons and their p-values.

**SOM5.** Pairwise comparisons and their p values.

| **Indicator** | **Pairwise comparison** | **Contrast** | **P-Value** |
| --- | --- | --- | --- |
| Break-off | Image vs Text | .70 | .00 |
|  | ImagePush vs Text | .68 | .00 |
|  | ImagePush vs Image | -.02 | 1.00 |
| Noncompliance | Image vs Text | 39.74 | .00 |
|  | ImagePush vs Text | 34.57 | .00 |
|  | ImagePush vs Image | -5.17 | .03 |
| Completion time | PC-Text vs PC-Image | 33.22 | .00 |
|  | PC-Text vs PC-ImagePush | 43.52 | .00 |
|  | S-Text vs S-Image | 25.61 | .00 |
|  | S-Text vs S-ImagePush | 26.37 | .00 |
|  | PC-Image vs S-Image | -5.01 | 1.00 |
|  | PC-ImagePush vs S-ImagePush | -14.55 | .00 |
|  | PC-Image vs PC-ImagePush | 10.29 | .05 |
|  | S-Image vs S-ImagePush | .75 | 1.00 |
| Easy | Image vs Text | -29.68 | .00 |
|  | ImagePush vs Text | -31.05 | .00 |
|  | ImagePush vs Image | -1.36 | .71 |
| Like | Image vs Text | -33.78 | .00 |
|  | ImagePush vs Text | -35.14 | .00 |
|  | ImagePush vs Image | -1.36 | .29 |

*Note:* Contrasts between APMs. In probabiltieis (0-100) for breakoff, noncompliance, *easy* and *like* and seconds for completion times. P-values computed using Bonferroni adjustments.
